# A substrate ambiguous enzyme facilitates genome reduction in an intracellular symbiont

**DOI:** 10.1186/s12915-014-0110-4

**Published:** 2014-12-20

**Authors:** Daniel RG Price, Alex CC Wilson

**Affiliations:** Department of Biology, University of Miami, Coral Gables, FL 33146 USA

**Keywords:** Bacteriocyte, Symbiosis, Endosymbiosis, Co-evolution, Gammaproteobacteria

## Abstract

**Background:**

Genome evolution in intracellular microbial symbionts is characterized by gene loss, generating some of the smallest and most gene-poor genomes known. As a result of gene loss these genomes commonly contain metabolic pathways that are fragmented relative to their free-living relatives. The evolutionary retention of fragmented metabolic pathways in the gene-poor genomes of endosymbionts suggests that they are functional. However, it is not always clear how they maintain functionality. To date, the fragmented metabolic pathways of endosymbionts have been shown to maintain functionality through complementation by host genes, complementation by genes of another endosymbiont and complementation by genes in host genomes that have been horizontally acquired from a microbial source that is not the endosymbiont. Here, we demonstrate a fourth mechanism.

**Results:**

We investigate the evolutionary retention of a fragmented pathway for the essential nutrient pantothenate (vitamin B5) in the pea aphid, *Acyrthosiphon pisum* endosymbiosis with *Buchnera aphidicola*. Using quantitative analysis of gene expression we present evidence for complementation of the *Buchnera* pantothenate biosynthesis pathway by host genes. Further, using complementation assays in an *Escherichia coli* mutant we demonstrate functional replacement of a pantothenate biosynthesis enzyme, 2-dehydropantoate 2-reductase (E.C. 1.1.1.169), by an endosymbiont gene, *ilvC*, encoding a substrate ambiguous enzyme.

**Conclusions:**

Earlier studies have speculated that missing enzyme steps in fragmented endosymbiont metabolic pathways are completed by adaptable endosymbiont enzymes from other pathways. Here, we experimentally demonstrate completion of a fragmented endosymbiont vitamin biosynthesis pathway by recruitment of a substrate ambiguous enzyme from another pathway. In addition, this work extends host/symbiont metabolic collaboration in the aphid/*Buchnera* symbiosis from amino acid metabolism to include vitamin biosynthesis.

**Electronic supplementary material:**

The online version of this article (doi:10.1186/s12915-014-0110-4) contains supplementary material, which is available to authorized users.

## Background

Extensive genome reduction in obligate endosymbiotic bacteria is a hallmark of intracellularity [1-3]. Recent genome sequencing of diverse bacterial endosymbionts of insects reveals ongoing symbiont genome reduction, resulting in small gene dense genomes that contain a subset of genes compared to their closest free-living relatives [4-7]. For example, the genome of the gammaproteobacterium *Buchnera* from the aphid, *Cinara cedri*, is only 416 kbp, approximately 11-fold smaller that its closest free-living relative *Escherichia coli* [4,8]. Currently, the smallest known bacterial genomes, containing fewer than 200 protein-coding genes, belong to insect associated obligate endosymbionts [6,7,9,10]. Eliminated genes include those that are either functionally redundant, or not essential for maintenance of the symbiosis [1-3]. Many of the genes evolutionarily retained in endosymbiont genomes are those required for biosynthesis of nutrients essential to the host. However, even endosymbiont essential nutrient biosynthesis pathways experience gene loss such that it is common to find fragmented, but evolutionary retained, pathways that appear incomplete and non-functional [11,12].

**Table 1:** **Pantothenate biosynthesis genes and enzyme activities**

***Buchnera and E*** **.** ***coli*** **vitamin B5 biosynthesis**				
Gene	*Buchnera* ID *#*	*E. coli* ID *#*	E.C *#*	Enzyme activity (abbreviated activity)
*ilvE*	x	EG10497	2.6.1.42	branched-chain amino-acid-transaminase (BCAT)
*ilvI*	BU226	EG10500	2.2.1.6	acetolactate synthase (ALS)
*ilvH*	BU225	EG10499	2.2.1.6	acetolactate synthase (ALS)
*ilvN*	x	EG10502	2.2.1.6	acetolactate synthase (ALS)
*ilvB*	x	EG10494	2.2.1.6	acetolactate synthase (ALS)
*ilvM*	x	EG10501	2.2.1.6	acetolactate synthase (ALS)
*ilvC*	BU599	EG10495	1.1.1.86	ketol-acid reductoisomerase (KARI)
*ilvD*	BU600	EG10496	4.2.1.9	dihydroxyacid dehydratase (DHAD)
*panB*	BU197	EG11675	2.1.2.11	ketopantoate hydroxymethltransferase (KPHMT)
*panE*	x	EG13271	1.1.1.169	ketopantoate reductase (KPR)
*panD*	x	EG11747	4.1.1.11	L-aspartate-α-decarboxylase (ADC)
*panC*	BU196	EG11746	6.3.2.1	pantothenate synthetase (PS)
***A. pisum*** **β-alanine biosynthesis**				
Gene	ACYPI ID *#*	LOC ID *#*	E.C *#*	Enzyme activity (abbreviated activity)
*DPD*	ACYPI004747	LOC100163680	1.3.1.2	dihydropyrimidine dehydrogenase (DPD)
*DHPase* ^a^	ACYPI002925	LOC100161725	3.5.2.2	dihydropyrimidinase (DHPase)
*BUP*	ACYPI003488	LOC100162330	3.5.1.6	beta-ureidopropionase (BUP)
*ADC*	ACYPI009960	LOC100169332	4.1.1.11	L-aspartate-α-decarboxylase (ADC)

^a^Alternative splice forms are annotated in National Center for Biotechnology Information (NCBI) *A. pisum* RefSeq (Acyr_2.0) gene models. Gene identification numbers (ID #) are from: *Buchnera* APS [12], *E. coli* K-12 MG1655 (ID# from EcoGene [37]) and *A. pisum* LSR1 [38]. Enzyme Commission number (E.C #) and enzyme activity are from the BRENDA enzyme information system [39].

Recent genome sequencing of holosymbionts (hosts + their symbionts) reveals that fragmented biosynthesis pathways can be completed by three processes: (1) complementation by host genes with overlapping function [13-15]; (2) complementation by genes encoded by another symbiont [4-6]; and (3) complementation by genes horizontally transferred to the host nuclear genome from other bacterial genomes [11,15]. All three of these processes can contribute to functional maintenance of metabolic pathways in a single endosymbiont and yet, even accounting for these three independent processes, some evolutionarily retained symbiont pathways remain fragmented making it unclear if they are functional [11,12].

A fourth process for completion of fragmented biosynthesis pathways was proposed by Shigenobu *et al.* [12] when they hypothesized that missing enzyme steps in fragmented *Buchnera* amino acid biosynthesis pathways are mediated by other (unidentified) *Buchnera* enzymes with overlapping activity [12,14]. In addition to well-defined primary roles within linear metabolic pathways, it is common for enzymes to catalyze side reactions [16-18]. Such multifunctional enzymes that are capable of catalyzing weak side reactions, exhibit either catalytic promiscuity, catalysis of a different reaction type on a single or range of substrates; or substrate ambiguity, catalysis of the same reaction type on a range of substrates [16-18]. Extraordinary levels of metabolic plasticity within organisms are facilitated by weak side reactions [19-24]. Importantly, weak side activities provide precursor catalytic activity for the evolution of new enzyme function [16-18,21,22]. It is not without precedent that multifunctional enzymes with overlapping activity ‘fill holes’ in fragmented essential metabolic pathways [25-27]. For example, the fragmented folate biosynthesis pathway from the obligate intracellular pathogen *Chlamydia* is functional because of the recruitment of promiscuous and adaptable enzymes from other biosynthetic pathways [25].

Here, working with the best-characterized holosymbiosis, that of the pea aphid *Acyrthosiphonpisum* with the gammaproteobacterium *Buchnera*, we reveal that the fragmented *Buchnera* pantothenate (vitamin B5) biosynthesis pathway can be completed using a *Buchnera* substrate ambiguous enzyme recruited from another pathway. We demonstrate using complementation assays in *E. coli* mutants that retention of a substrate ambiguous enzyme in the massively reduced genome of *Buchnera* facilitates the collaborative biosynthesis of pantothenate (vitamin B5) in the *A. pisum*/*Buchnera* holosymbiont.

## Results and discussion

### *Buchnera* has a fragmented but evolutionary retained pantothenate biosynthesis pathway

**Figure 1:**
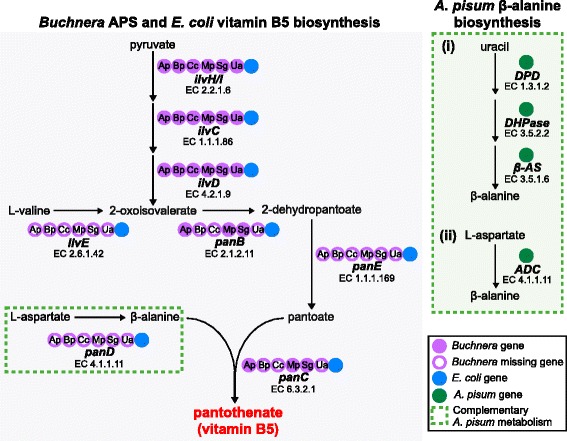
**Comparative analysis of the** ***Buchnera*** **pantothenate biosynthesis pathway and complementary** ***A. pisum*** **β-alanine biosynthesis pathways.** Metabolic pathways were reconstructed using KEGG pantothenate biosynthesis pathway from *E. coli* K-12 (eco00770) and from *Buchnera* from host aphids that included: *A. pisum* (Ap, buc00770), *B. pistaciae* (Bp, bab00770), *C. cedri* (Cc, bcc00770), *M. persicae* (Mp, [30]), *S. graminum* (Sg, bas00770) and *U. ambrosiae* (Ua, bua00770) [34,35]. Complementary β-alanine biosynthesis pathways of *A. pisum* (api00410) [34,35] are shown. The presence/absence of biosynthesis genes in the *Buchnera, E. coli* and *A. pisum* genomes is indicated. Full details of *A. pisum*, *Buchnera* and *E. coli* gene identifiers and enzyme activities are provided in Table 1. KEGG, *Kyoto Encyclopedia of Genes and Genomes*.

Compared to *E. coli*, the obligate endosymbiont *Buchnera* from the pea aphid, *A. pisum*, has a fragmented pantothenate biosynthesis pathway that is missing *panD*, *panE* and *ilvE* (Figure 1). Gene identifiers and enzyme activities are provided in Table 1. *Buchnera* with fully sequenced genomes fall into two groups: (1) those from species such as *Baizongia pistaciae*, *C. cedri* and *Uroleucon ambrosiae* that have lost the whole pantothenate biosynthesis pathway [4,28,29]; and (2) those from species such as *A. pisum*, *Myzus persicae* and *Schizaphis graminum* that retain identically fragmented pathways characterized by loss of *panD*, *panE* and *ilvE* [12,30,31] (Figure 1). Evolutionary retention by multiple *Buchnera* lineages of identically fragmented pantothenate biosynthesis pathways that include two genes (*panB* and *panC*) that only function in pantothenate biosynthesis, led us to hypothesize that the pantothenate biosynthesis pathway in these species is functional.

**Figure 2:**
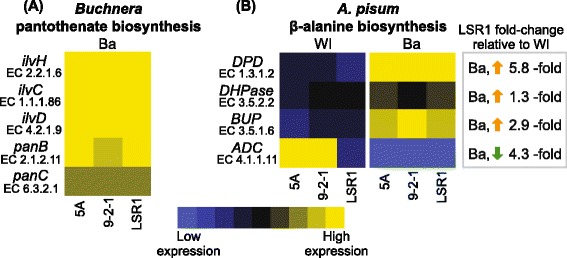
**Quantitative expression analyses of** ***Buchnera and A. pisum*** **pantothenoate biosynthesis genes.** Expression profiles for **(A)** *Buchnera* pantothenate biosynthesis genes and **(B)** complementary *A. pisum* β-alanine biosynthesis genes were generated for three independent *A. pisum* lineages (5A, 9-2-1 and LSR1). Expression of *Buchnera* genes in bacteriocytes (Ba) was normalized to *atpE* (BU003) while expression of *A. pisum* genes in whole insects (WI), bacteriocyte (Ba) and gut (G) tissues was normalized to *GAPDH* (ACYPI009769). Relative abundance of each gene was compiled into a heat map showing high expression (yellow) to low expression (blue). The box shows gene expression fold-change in *A. pisum* LSR1 bacteriocyte (Ba) tissues relative to whole insect (WI) expression. Fold-change was calculated according to [36]; for each aphid lineage *n* = 3.

To begin the investigation of our hypothesis that evolutionary retention of identically fragmented pantothenate biosynthesis pathways indicates retention of pantothenate biosynthesis capacity we characterized expression of *ilvH*, *ilvC*, *ilvD*, *panB* and *panC* in the specialized aphid bacteriocyte cells that house *Buchnera*. All five of these *Buchnera* genes were highly expressed in *A. pisum* bacteriocytes and consistently highly expressed across three genetically independent *A. pisum* lines (Figure 2A). Functionality of these fragmented pathways requires (1) host supply of β-alanine and (2) complementation of *panE* encoded 2-dehydropantoate 2-reductase (E.C. 1.1.1.169).

### *A. pisum* synthesizes β-alanine via the uracil degradation pathway in bacteriocytes

*A. pisum* and other insects synthesize β-alanine via two main pathways: (1) the uracil degradation pathway [32] and (2) via decarboxylation of L-aspartate [33] (Figure 1). Gene identifiers and enzyme activities are provided in Table 1. While synthesis of β-alanine is evolutionarily conserved in the major insect orders (*Kyoto Encyclopedia of Genes and Genomes* (KEGG) pathway map 00410), we propose that β-alanine biosynthesis in *A. pisum* is now additionally required to supply *Buchnera*.

Gene expression analysis demonstrates that β-alanine biosynthesis via the *A. pisum* uracil degradation pathway is upregulated in bacteriocytes relative to whole insect expression (Figure 2B). Consistent with uracil degradation being the major β-alanine biosynthesis route in bacteriocytes, the alternate pathway that synthesizes β-alanine via decarboxylation of L-aspartate is down regulated in bacteriocytes (Figure 2B). Aspartate decarboxylase (ADC) is 4.3-fold down in LSR1 bacteriocytes, 25-fold down in 5A bacteriocytes and 100-fold down in 9-2-1 bacteriocytes relative to whole insect expression.

### *Buchnera ilvC* complements *panE* to complete the pantothenate biosynthesis pathway in *A. pisum*

Given host supply of β-alanine, functional pantothenate biosynthesis in *Buchnera* with the fragmented pantothenate biosynthesis pathway requires complementation of lost *panE* encoded 2-dehydropantoate 2-reductase (E.C. 1.1.1.169)*.* Previous work in *Salmonella typhimurium* [40] and *E. coli* [41] has shown that *ilvC*, a gene encoding ketol-acid reductoisomerase (E.C. 1.1.1.86) that mediates reactions in the biosynthesis of the essential amino acids isoleucine and valine and production of 2-oxoisovalerate from pyruvate in pantothenate biosynthesis, also has limited 2-dehydropantoate 2-reductase (E.C. 1.1.1.169) activity. While ilvC can reduce 2-dehydropantoate to pantoate, the activity of ilvC in *S. typhimurium* to function in pantothenate biosynthesis is 20-fold lower than its activity as ketol-acid reductoisomerase [40] and so low in *E. coli* that the *ilvC* knockout mutant was indistinguishable from wild-type K12 in its reduction of 2-dehydropantoate to pantoate [41]. However, given that generation of *E. coli* pantoate auxotrophs requires generation of *panE*, *ilvC* double mutants, we hypothesized that *Buchnera ilvC* has sufficient 2-dehydropantoate 2-reductase activity and, thereby, critically maintains functional pantothenate biosynthesis in *Buchnera* that retain fragmented pantothenate biosysnthesis pathways.

**Figure 3:**
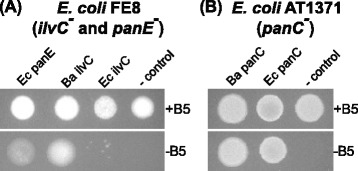
**Functional complementation of** ***E. coli*** **pantothenate biosynthesis auxotrophs with** ***Buchnera*** **pantothenate biosynthesis genes. (A)** *E. coli* FE8 cells (*ilvC* ^*-*^ and *panE* ^*-*^) were transformed with expression plasmids containing *E. coli panE*, *Buchnera ilvC*, *E. coli ilvC* and negative control pUC19 empty vector (- control). For complementation assays 5 μl of cells (from *OD*_600_ = 1.0) was plated on minimal media plates with pantothenate (+B5, non-selective) and without pantothenate (-B5, selective). Recovery of *E. coli* mutant growth was assessed after four days at 26°C. **(B)** *E. coli* AT1371 cells (*panC* ^-^) were transformed with expression plasmids containing *Buchnera panC, E. coli panC* and negative control pUC19 empty vector (- control). Complementation assays were completed as described in **(A)**.

To test the hypothesis that *Buchnera ilvC* has 2-dehydropantoate 2-reductase activity sufficient to complete pantoate synthesis we utilized a 2-dehydropantoate 2-reductase negative strain of *E. coli* (strain FE8: *ilvC*^-^ and *panE*^-^ [41]) and functionally complemented *E. coli* FE8 with *Buchnera ilvC* (Figure 3A)*.* When cells were plated on selective plates (minimal media plates, supplemented with L-isoleucine and 3-methyl-2-oxobutanoic acid to complement deletion of *ilvC* but without pantothenate supplementation), rescue of *E. coli* FE8 was only achieved by complementation with *E. coli panE* and *Buchnera ilvC* (Figure 3A). In contrast, under selective conditions, very limited growth of FE8 cells transformed with *E. coli ilvC* was observed, suggesting that *E. coli ilvC* does not contain sufficient 2-dehydropantoate 2-reductase activity to rescue 2-dehydropantoate 2-reductase negative *E. coli* cells, a result consistent with the earlier work of Elischewski *et al*., [41]. As a negative control, *E. coli* FE8 was transformed with pUC19 empty expression vector. As expected, these pUC19 empty vector transformed cells did not grow without pantothenate supplementation (Figure 3A). When grown in non-selective conditions (minimal media plates with L-isoleucine, 3-methyl-2-oxobutanoic acid and pantothenate supplementation) FE8 cells transformed with *E. coli panE*, *Buchnera ilvC*, *E. coli ilvC* and pUC19 empty expression vector (negative control) established robust colonies (Figure 3A).

Functional complementation assays were also completed to test the pantoate-β-alanine ligase (E.C. 6.3.2.1) activity of *Buchnera panC. Buchnera panC* shares only 48% amino acid sequence identity with *E. coli panC*. Despite this high level of sequence divergence, *Buchnera panC* retains pantoate-β-alanine ligase activity (Figure 3B). Both *Buchnera* APS *panC and E. coli panC* (positive control) functionally rescue *E. coli* AT1371 (*panC*^-^ strain) when grown on a minimal media without pantothenate supplementation. Negative control *E. coli* AT1371 transformed with pUC19 empty expression vector was unable to grow on minimal media without pantothenate supplementation. With pantothenate supplementation, growth of AT1371 transformed with *Buchnera panC*, *E. coli panC* and negative control cells is observed (Figure 3B).

### Loss of *Buchnera ilvE* facilitates holosymbiont integration

Loss of *Buchnera ilvE* (encoding branched-chain-amino-acid transaminase, E.C. 2.6.1.42) is not critical to the integrity of the pantothenate biosynthesis pathway in *Buchnera* because *Buchnera* with fragmented pathways retain *ilvH/I*, *ilvC* and *ilvD* and are predicted to synthesize 2-oxoisovalerate from pyruvate (Figure 1). However, the loss of *Buchnera ilvE* has important coevolutionary implications for the biosynthesis of the branched chain amino acids (isoleucine, leucine and valine). Symbiont loss of *ilvE* was first reported in *Buchnera* APS from *A. pisum* [12] a decade before the genome of *A. pisum* was published [38]. While Shigenobu *et al.,* [12] had speculated that *Buchnera* APS likely utilized the activity of another transaminase to complete branched chain amino acid biosynthesis, members of the Pea Aphid Genome Consortium offered an alternate explanation [42], that *Buchnera* loss of *ilvE* encoded branched-chain-amino-acid transaminase is complemented by the host genome encoded branched-chain-amino-acid transaminase (BCAT, ACYPI008371, [42]). Recently, the collaborative role of BCAT in the biosynthesis of leucine, one of the three branched chain amino acids, has been confirmed experimentally [43]. Identical patterns of holosymbiont coevolution involving symbiont loss of *ilvE* have likely also coevolved in the citrus mealybug, *Planococcus citri* [11], and the whitefly, *Bemisia tabaci* [31].

### Host/symbiont metabolic collaboration and a substrate ambiguous enzyme facilitate maintenance of functional pantothenate biosynthesis pathways in some *Buchnera* lineages

**Figure 4:**
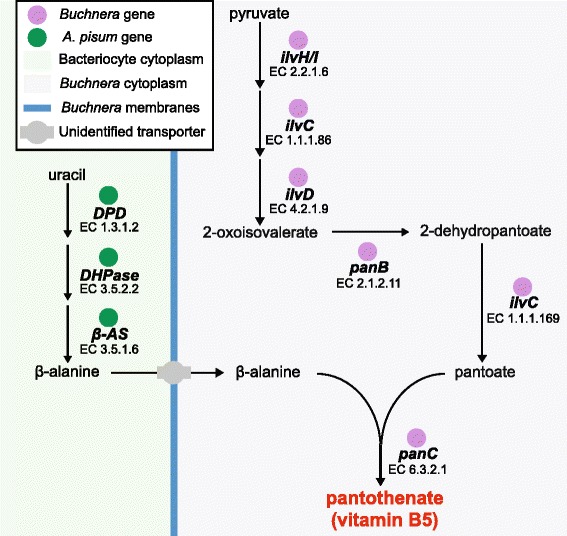
**Pantothenate (vitamin B5) biosynthesis in the** ***A. pisum*** **/** ***Buchnera*** **holosymbiont.**

On the basis of holosymbiont metabolic pathway analysis, gene expression analysis and complementation tests in *E. coli* pantothenate biosynthesis mutants, we propose a model of collaborative pantothenate biosynthesis that critically depends on host supply of β-alanine, and the substrate ambiguous activity of ketol-acid reductoisomerase (E.C. 1.1.1.86) encoded by *ilvC* as 2-dehydropantoate 2-reductase (E.C. 1.1.1.169) (Figure 4).

*A. pisum* synthesize β-alanine in bacteriocytes via the uracil degradation pathway (Figure 4). To complete pantothenate biosynthesis, β-alanine is transported from the bacteriocyte cytoplasm to *Buchnera* via a currently unidentified transporter (Figure 4). In *Buchnera’s* closest free-living relative *E. coli*, β-alanine is actively transported via amino acid transporter *cycA* [44]. However, due to extensive gene loss, *Buchnera* has lost almost all substrate specific transporters, including *cycA* [12,45]. Our current working hypothesis, as previously proposed by Shigenobu *et al*., [12], is that *Buchnera* utilizes a flagellum-derived general substrate transport system for import of essential nutrients, including β-alanine.

Notably, we demonstrate that *panE* encoding 2-dehydropantoate 2-reductase has been lost from *Buchnera’s* genome and functionally replaced with *ilvC*, an enzyme that encodes both ketol-acid reductoisomerase (E.C. 1.1.1.86) and 2-dehydropantoate 2-reductase (E.C. 1.1.1.169) activity. The gene product of *ilvC* functions as a substrate ambiguous reductase in branched chain amino acid biosynthesis and pantothenate biosynthesis. Functional replacement of *panE* encoded ketopantoate reductase with a substrate ambiguous *ilvC* encoded ketopantoate reductase is not without precedent since loss of *panE* and functional replacement by *ilvC* has previously been demonstrated in the gram-positive soil bacterium *Corynebacterium glutamicum* [46]. In the context of holosymbiont evolution, we hypothesize that retention of multifunctional endosymbiont enzymes facilitates genome reduction while maintaining the functional integrity of essential biosynthetic pathways, a process that facilitates genome reduction but results in increased functional complexity of symbiont-retained genes. Consistent with our hypothesis, in genome-wide analyses, it has recently been demonstrated that bacterial species with small, reduced genomes have increased protein functional complexity, leading the authors to suggest that an increase in functional complexity of retained genes in small genomes compensates for gene losses [47].

## Conclusions

There are currently four mechanisms that facilitate retention of metabolic functionality concomitant with gene loss in the primary endosymbionts of insects. All four mechanisms can operate within a single genome and all four contribute to explaining why fragmented and disconnected biosynthesis pathways, that on first observation appear incomplete and non-functional, are retained across diverse obligate endosymbiont taxa. As more holosymbiont genomes become available, metabolic reconstruction of obligate intracellular symbionts must consider a complex evolutionary patchwork that comprises host metabolic complementarity, symbiont replacement, horizontal transfer of complementary biosynthesis genes to the host genome and now also protein multifunctionality in endosymbionts, all of which contribute to the maintenance of endosymbiont metabolic function.

## Methods

### Aphid culture

Parthenogenetic females of the pea aphid *A. pisum*, lineage LSR1 [38], 5A and 9-2-1 [48] were maintained on pre-flowering *Vicia faba* cv. The Sutton. All aphids were kept at 20°C in a long day regime of 16 hours of light and 8 hours of dark.

### Comparative analysis of vitamin B5 biosynthesis

Metabolic pathways for pantothenate (vitamin B5) biosynthesis were analyzed using the KEGG pathway database [34,35]. These included the pantothenate biosynthesis pathways from *E. coli* K-12 MG1655 (eco00770) and *B. aphidicola* from host aphids, including: *A. pisum* (*Buchnera* Ap, buc00770 [12]), *B. pistaciae* (*Buchnera* Bp, bab00770 [28]), *C. cedri* (*Buchnera* Cc, bcc00770 [4]), *M. persicae* (*Buchnera* Mp [30]), *S. graminum* (*Buchnera* Sg, bas00770 [31]) and *U. ambrosiae* (Ua, bua00770 [29]). The complementary *A. pisum* β-alanine metabolism pathway was identified in *A. pisum* LSR1 (api00410 [38]).

### Real-time quantitative RT-PCR

Real-time quantitative PCR (QPCR) was used to compare *A. pisum* and *Buchnera* pantothenate biosynthesis gene expression in whole adult female aphids and isolated bacteriocytes using 2^-ΔΔ*C*T^ methodology [36]. Methods are identical to those described in detail by [49]. Primers were designed using Primer3Plus software for real-time PCR [50], and specificity checked using Primer-BLAST against NCBI *A. pisum* RefSeq gene models (primer sequences are shown in Additional file 1: Table S1). For comparative gene expression analysis, all QPCR primers had amplification efficiencies >90% and <105%. *A. pisum* gene expression was normalized to *glyceraldehyde-3-dehydrogenase* (*GAPDH*, ACYPI009769) and *Buchnera* gene expression was normalized to *atpE ATP synthase F0F1 subunit C* (*atpE*, BU003). The normalized expression value for each gene was compiled into a heat map (*z*-score x 10; *z* = (each value - average)/standard deviation). Yellow: *z* >0, blue: *z* <0, black: *z* = 0.

### *ilvC, panC and panE* expression constructs

Full-length *Buchnera ilvC* (BU599) and *panC* (BU196) coding sequences were amplified from *Buchnera* LSR1 gDNA, and full-length *E. coli ilvC* (EG10495), *panC* (EG11746) and *panE* (EG13271) coding sequences were amplified from *E. coli* K-12 MG1655 gDNA; all coding sequences were amplified using Phusion high-fidelity DNA polymerase (Thermo Scientific, Waltham, MA, USA). Primers were designed to incorporate restriction enzyme sites to allow directional cloning of *ilvC*, *panC* and *panE* coding sequences into pUC19, in frame and downstream of the *lacZ* start codon; thus, generating pUC19 expression constructs containing *ilvC, panC* and *panE* coding sequences under the control of the *lacZ* promoter (primer sequences are shown in Additional file 1: Table S1). Amplification reactions were completed on a Mastercycler PCR system (Eppendorf, Hauppauge, NY, USA) with the following conditions: 98°C for 30 seconds, followed by 30 cycles at 98°C for 10 seconds, 55°C for 30 seconds and 72°C for 1 minute. Amplified coding sequences were digested with either HindIII and BamHI, or BamHI and EcoRI (see Additional file 1: Table S1) and cloned into the respective sites of pUC19. Expression constructs containing *Buchnera* and *E. coli ilvC* coding sequences were named pBUC-*ilvC* and pECO-*ilvC*, respectively. Expression constructs containing *Buchnera* and *E. coli panC* coding sequences were named pBUC-*panC* and pECO-*panC*, respectively. The expression construct containing *E. coli panE* coding sequence was named pECO-*panE*. All expression constructs were fully sequenced using Sanger sequencing in standard Big Dye terminator v3.1 reactions (Life Technologies, Green Island, NY, USA). Reaction products were analyzed on 3130xl genetic analyzer (Life Technologies), and sequence data assembled into contiguous sequences using Sequencher v4.9 (Gene Codes Corp., Ann Arbor, MI, USA).

### Functional complementation of *E. coli* auxotrophs

Functional complement experiments were carried out using the *E. coli ilvC*/*panE* double mutant FE8 (*ilvC::aacC1, panE::tetA*; [41]), obtained as a kind gift from Jörn Kalinowski (Institute for Genome Research and Systems Biology, Bielefield University, Germany); and *E. coli panC* mutant AT1371 (F^-^, *panC4*, *Δ(gpt-proA)62*, *lacY1*, *tsx-29*, *glnV44*(AS), *galK2*(Oc), *λ*^*-*^, *Rac-0*, *hisG4*(Oc), *rfbC1*, *xylA5*, *mtl-1*, *argE3*(Oc), *thiE1* [51,52]), obtained from the Yale University *E.coli* Genetic Stock Center (CGSC, Yale University, New Haven, CT, USA.).

*E. coli* FE8 (*ilvC*^-^ and *panE*^-^) cells were transformed with plasmids pBUC-*ilvC*, pECO-*ilvC,* pECO-*panE* and pUC19 empty vector (negative control) according to [53]. Transformants were grown overnight in Luria-Bertani (LB) media with 50 μg/ml ampicillin, 15 μg/ml gentamicin and 10 μg/ml tetracycline and washed twice in sterile distilled water and finally resuspended to an attenuance of *OD*_600_ = 1.0. Functional complementation of *E. coli* FE8 pantothenate auxotrophs was carried out by plating aliquots (5 μl) of cell suspensions on M63 minimal media agar plates [54] containing 13.6 g/L KH_2_PO_4_ (pH 7.0), 2 g/L (NH_4_)_2_SO_4,_ 0.4% (w/v) glycerol, 0.25 g/L MgSO_4_.7H_2_O, 50 mg/ml L-isoleucine, 50 mg/ml 3-methyl-2-oxobutanoic acid (Acros Organics, Waltham, MA, USA), 0.5 mg/L FeSO_4_.7H_2_O, 0.5 mg/L thiamine, 1.5% (w/v) agar, 50 μg/ml ampicillin, 15 μg/ml gentamicin and 10 μg/ml tetracycline. Aliquots of cell suspensions were also plated on M63 minimal media agar plates supplemented with 50 μg/ml D-pantothenic acid (non-selective) to assess cell viability. *E. coli* FE8 cell growth on selective plates (without vitamin B5) and non-selective plates (with vitamin B5) was assessed after four days of growth at 26°C.

*E. coli* AT1371 (*panC*^-^) cells were transformed with plasmids pBUC-*panC*, pECO-*panC* (positive control) and pUC19 empty vector (negative control) according to [53]. Transformants were grown overnight in LB media with 50 μg/ml ampicillin and washed twice in sterile distilled water and finally resuspended to an attenuance of OD600 = 1.0. Functional complementation of AT1371 pantothenate auxotrophs was carried out by plating aliquots (5 μl) of cell suspensions on M63 minimal media agar plates [54] containing 13.6 g/L KH_2_PO_4_ (pH 7.0), 2 g/L (NH_4_)_2_SO_4,_ 0.4 % (w/v) glycerol, 0.25 g/L MgSO_4_.7H_2_O, 0.5 mg/LFeSO_4_.7H_2_O, 0.5 mg/L thiamine, 1.5% (w/v) agar and 100 μg/ml ampicillin. Aliquots of cell suspensions were also plated on M63 minimal media agar plates supplemented with 50 μg/ml D-pantothenic acid (non-selective) to assess cell viability. *E. coli* FE8 cell growth on selective plates (without pantothenate) and non-selective plates (with pantothenate) was assessed after four days of growth at 26°C.

## Additional file

Additional file 1: Table S1. Pantothenate biosynthesis primers. Notes: ^*^Alternative splice forms are annotated in NCBI *A. pisum* RefSeq (Acyr_2.0) gene models. QPCR primers were designed to amplify all splice forms. Restriction enzyme (RE) sites for forward (fwd) and reverse (rev) expression construct primers are shown in bold.
